# Why are a quarter of all cancer deaths in south-east England registered by death certificate only? Factors related to death certificate only registrations in the Thames Cancer Registry between 1987 and 1989.

**DOI:** 10.1038/bjc.1995.125

**Published:** 1995-03

**Authors:** A. M. Pollock, N. Vickers

**Affiliations:** Department of Public Health Sciences, St George's Hospital Medical School, London, UK.

## Abstract

This paper describes the results of a study set up to investigate factors associated with the high proportions of 'death certificate only' registrations (DCOs) for all cancers registered in south-east England between 1987 and 1989 and to identify those which might be subject to registry intervention. DCOs as a proportion of all registrations (n = 162,131) were analysed by age, sex, district of residence, place of death and survival. DCO registration ratios (standardised for age and sex) were then derived for each of the 56 districts in the Thames Regions. A multiple logistic regression model was generated to estimate the effect of age at diagnosis, tumour survival and patient sex on final source of registration. To minimise the number of dummy variables needed, each of the 56 districts was ranked into quartiles: quartile 1 contained the 14 districts with the lowest age- and sex-standardised ratios for DCO registrations and quartile 4 comprised the 14 districts with the highest DCO ratios. Final source of registration was treated as a binomial trial (case notes or death certificates). The significance of associations was measured using the deviance difference as an approximate chi-square statistic. The effect of each variable on source of registration was estimated as an odds ratio. Interaction terms were also fitted. To estimate the effect of place of death on DCO registrations, a second model was generated for deceased patients only (n = 98,455, adding 'place of death' to the list of explanatory variables already used. A further interaction term was fitted to account for interaction between place of death and district quartile of residence. Around 24% of all patient deaths were registered as DCOs by the Thames Cancer Registry between 1987 and 1989. Of these, 40.9% died in an acute NHS hospital setting, 37.1% died at home, 10.4% died in hospices and 3.4% died in non-NHS hospitals. Increasing age, decreasing survival, district of residence and place of death were positively associated with death certificate registrations. The district effect was sustained in the regression model with significant positive associations shown for DHA quartile of residence. In the deceased group of patients, both district of residence and place of death were independent predictors of DCOs. Death occurring outside the acute NHS hospital setting increased the odds of being a DCO within and across district quartiles. DCOs could be reduced by better case ascertainment in some districts.(ABSTRACT TRUNCATED AT 400 WORDS)


© 1995 Stockton Press All rights reserved 0007–0920/95 $9.00

### Why are a quarter of all cancer deaths in south-east England registered by death certificate only? Factors related to death certificate only registrations in the Thames Cancer Registry between 1987 and 1989

AM Pollock and N Vickers

*Department of Public Health Sciences, St George's Hospital Medical School, London SW17 0RE, UK.*

**Summary** This paper describes the results of a study set up to investigate factors associated with the high proportions of 'death certificate only' registrations (DCOs) for all cancers registered in south-east England between 1987 and 1989 and to identify those which might be subject to registry intervention. DCOs as a proportion of all registrations ($n$ = 162 131) were anlaysed by age, sex, district of residence, place of death and survival. DCO registration ratios (standardised for age and sex) were then derived for each of the 56 districts in the Thames Regions. A multiple logistic regression model was generated to estimate the effect of age at diagnosis, tumour survival and patient sex on final source of registration. To minimise the number of dummy variables needed, each of the 56 districts was ranked into quartiles: quartile 1 contained the 14 districts with the lowest age- and sex-standardised ratios for DCO registrations and quartile 4 comprised the 14 districts with the highest DCO ratios. Final source of registration was treated as a binomial trial (case notes or death certificates). The significance of associations was measured using the deviance difference as an approximate chi-square statistic. The effect of each variable on source of registration was estimated as an odds ratio. Interaction terms were also fitted. To estimate the effect of place of death on DCO registrations, a second model was generated for deceased patients only ($n$ = 98 455, adding 'place of death' to the list of explanatory variables already used. A further interaction term was fitted to account for interaction between place of death and district quartile of residence. Around 24% of all patient deaths were registered as DCOs by the Thames Cancer Registry between 1987 and 1989. Of these, 40.9% died in an acute NHS hospital setting, 37.1% died at home, 10.4% died in hospices and 3.4% died in non-NHS hospitals. Increasing age, decreasing survival, district of residence and place of death were positively associated with death certificate registrations. The district effect was sustained in the regression model with significant positive associations shown for DHA quartile of residence. In the deceased group of patients, both district of residence and place of death were independent predictors of DCOs. Death occurring outside the acute NHS hospital setting increased the odds of being a DCO within and across district quartiles. DCOs could be reduced by better case ascertainment in some districts. Quality assurance measures should include monitoring DCO rates by district, site and registry. This would enable cancer registries to identify districts and tumours at high risk of DCO registration and enable the process of registrations and retrospective follow-up to be scrutinised. Changing patterns of treatment and terminal care may make case ascertainment and registration more difficult for registry staff in the future, although the minimum contract data set should assist in this. The current trends to shorten lengths of stay and increase day case and out-patient treatment could adversely affect registration and case ascertainment, especially if fewer people die in hospital.

**Keywords**: cancer registration; cancer survival; death certificates; England and Wales

Routine statistics on national cancer incidence and survival are derived by the Office of Population Censuses and Surveys (OPCS) from the 12 cancer registries of England and Wales (OPCS and Cancer Research Campaign, 1981; OPCS, 1993). They are used to monitor national trends in cancer incidence and survival and as a baseline for epidemiological studies and health services research (Swerdlow, 1986). In a recent study we showed that the quality and reliability of registrations are affected by the proportion of death certificate only registrations (DCOs) held by the registry (Pollock and Vickers, 1994). DCOs are registrations based on death certificate information alone (Jensen *et al.*, 1991).

Although OPCS holds no national data on DCOs, the annual reports of the Thames Cancer Registry (TCR) indicate that, between 1987 and 1989, DCOs constituted 23.8% of all registrations (Thames Cancer Registry, 1992). Since 1983, a rapid increase has taken place in Thames DCO rates. The registry has explained this rise by reference to the decision taken in 1983 (for financial reasons) not to follow up patients dying at home (Thames Cancer Registry, 1992). A second reason for the high rate was the amalgamation of the North Thames Regions, which only became part of the territory covered by the TCR in 1985: the greatest concentration of DCOs was found in the North Thames Regions. Thames DCO proportions compared unfavourably with those reported by registries elsewhere in England and Wales of between 1.6% and 13.8% (England and Wales registries, personal communications). Since 1992, the TCR has been attempting to retrieve data on DCO cases (including those patients dying at home) through family health services authorities (FHSAs).

High DCO proportions may bias the calculation of incidence, survival and treatment rates through inaccurate coding of tumour site, date of diagnosis or cause of death (Percy *et al.*, 1981; Chow and Devesa, 1992; Pollock and Vickers 1994*a*) or through loss of data (Silman and Evans, 1981; Swerdlow, 1986; Wilson *et al.*, 1992; Pollock and Vickers 1994*b*). Since it is usually impossible to confirm a date of diagnosis for DCOs, they are excluded from survival analysis.

The TCR covers a population of 13.8 million residents within the four regional health authorities (RHAs) and 56 district health authorities (DHAs) in south-east England. It contributes nearly a third of all cases to the national data. Its main sources of data for registration are clinical records and death certificates supplied by OPCS. If, on the basis of death certificate information, a place of treatment can be traced, the registry attempts to obtain confirmation of the diagnosis from the hospitals nearest to the place of death, the certifying physician or coroner. This process is called retrospective follow-up. If a place of treatment is traced, the registry attempts to supplement death certificate data with case note data, using peripatetic research clerks. These clerks are trained by the registry to abstract details from case notes in

Correspondence: AM Pollock
Received 22 March 1994; revised 16 August 1994; accepted 27 October 1994

medical records departments. If no further information is obtained on a death certificate registration, the registration is termed a death certificate only registration (DCO) (Jensen *et al.*, 1991).

DCOs have been linked to 'the efficiency of initial cancer registration procedures and the assiduousness with which different registries and registry clerks seek confirmatory evidence of the diagnosis of such cancers' (Wilson *et al.*, 1992). Although there are no previous studies of factors associated with DCOs, higher proportions of DCOs might be expected among patients diagnosed post mortem, patients dying at home, patients not receiving active treatment, patients with short survival and patients treated at centres which do not liaise with cancer registries (e.g. some private institutions). This paper describes the results of a study set up to investigate factors related to the high rates of DCOs observed in the Thames Cancer Registry (TCR) between 1987 and 1989 and to identify those which might be subject to registry intervention. In particular, we wished to ascertain whether place of death, district of residence and survival time affected DCO rates.

### Methods

All patients resident in the four Thames regional health authorities diagnosed as having cancer between 1987 and 1989 were identified from the Thames Cancer Registry ($n = 162\,131$). Death certificate only registrations (DCOs) as a proportion of these registrations were calculated by age, sex, place of death and tumour site.

DCO registration ratios for all tumour sites combined were calculated using indirect standardisation (as for standardised mortality ratios) for each of the 56 district health authorities in the four south Thames regions (adjusted for age and sex). Ninety-nine per cent confidence intervals were calculated using the method described by Gardner and Altman (1989). The significance of variations was further measured using the chi-square test for heterogeneity described by Breslow and Day (1987). Each district DCO ratio was ranked and assigned to quartiles such that quartile 1 contained the 14 districts with the lowest DCO ratios and quartile 4 comprised the 14 districts with the highest DCO ratios.

It might be expected that higher proportions of DCOs will be found in districts with a higher incidence of poor survival cancers, e.g. lung cancer. To adjust for district differences in the ratio of high survival to low survival cancers, 2 year relative survival rates were calculated for the 15 most common primary tumour sites in each sex for all registrations excluding DCOs ($n = 104\,370$), using the Hakulinen computer program (Hakulinen *et al.*, 1988). Each case, including DCOs ($n = 134\,927$), was then assigned a survival coefficient corresponding to the relative survival rate associated with the primary tumour site. Relative survival is the ratio of the survival observed in a group of cancer patients to the survival expected if they were only subject to the general (all-cause) mortality in a standard population. The standard population used in this study was the England and Wales population.

To test the significance of the association between district of residence and DCO registration while controlling for age at diagnosis, tumour survival and patient sex, a (backwards stepwise) multiple logistic regression model was generated. The model used DHA quartiles rather than DHAs (to minimise the number of dummy variables needed). Since our measurement of survival is a proxy for tumour site (each site has a different survival coefficient) we did not include tumour site as a separate variable. Final source of registration (case notes or death certificates) was the outcome variable. Two models were generated. The first included surviving patients in order to obtain a wider range of survival times and ages at diagnosis and thus estimate the effect of these variables. The second model examined deceased patients only.

The significance of associations was measured using the deviance difference (namely the change in deviance arising from the inclusion of each variable) as an approximate chi-square statistic. All variables were modelled as categorical variables except age at diagnosis and survival coefficient (which were modelled as continuous variables). The increased risk associated with each variable for DCO registration is measured in odds ratios. The modelling was carried out using the 'proc logist' procedure in the SAS computer program (SAS Institute, 1990).

To establish whether district of residence was a proxy measure for place of death, a second model was generated for deceased patients only ($n = 98\,455$), adding 'place of death' to the list of explanatory variables already used.

In both models, interactions between all variables were tested for and the final models include significant interactions.

### Results

### *Baseline analysis*

*Age and sex* DCOs accounted for 23.8% of all registrations. Table I shows that death certificates as a proportion of all registrations increased with age for both men and women. Among men, the number of DCO registrations rose from 9.1% in the under-40s to 32.4% in the 75 and over age group. Among women, the increase was even greater: DCOs accounted for 7.0% in the under-40s and 34.8% in the 75 and over age group. The odds of being registered as a DCO at age 65–74, relative to a male patient aged under 40, were 2.91 for men and 2.73 for women. These rose to 4.77 and 5.31 respectively at age 75 and over.

*Survival* DCO proportions and 2 year relative survival rates for non-DCO registrations for the 15 most common cancers in men and women are shown in Tables II and III for three survival categories: good, moderate and poor. DCO proportions tend to increase with more aggressive tumours, with a few exceptions. Good-survival tumours, such as testis and skin cancer, have lower proportions of DCOs (less than 10%). In the moderate survival category, DCO proportions ranged from 17% to 26%, except for multiple myeloma, where DCOs made up 33% of all registrations. This figure is similar to those for cancers associated with poor survival, such as lung, stomach and pancreas. Breast cancer, which has moderate to good survival, had 16% DCO registration. The proportion of DCOs increased significantly with decreasing survival in both men and women (Pearson correlation coefficient, $P < 0.0001$).

*District effect* Figure 1 shows the distribution of standardised ratios for DCO registrations (adjusted for age and sex) for each DHA, with 99% confidence intervals. The chi-square test for heterogeneity showed significant variations in district ratios ($\chi^2$ for heterogeneity = 1371.4, $P < 0.0001$).

*Place of death* The distribution of deaths and DCO registrations by place of death and quartile of residence is shown in Table IV: 48.3% of deaths occurred in an acute NHS hospital, 28% and 12% of all deaths arise at home and in hospices respectively and there is variation across the four quartiles. Of the DCO registrations, 40.9% die in an acute NHS hospital, 37.1% at home and 10.4% in hospices; again the proportions vary across the quartiles.

### *Multiple logistic regression models*

Table V lists the significance of associations for each variable with registration by DCO for all patients (alive and deceased combined). The odds ratios presented for survival represents the change in risk (of registration by DCO) associated with a 1% increase in 2 year relative survival rates. The odds ratios presented for age at diagnosis represent the change in risk associated with each added year at diagnosis. Table VI tests for the significance of associations between the same variables on deceased patients only (adding place of death). In both cohorts, all variables – DHA quartile, survival time, age, sex and (in the deceased patients' cohort) place of death – were strongly associated with DCO registration ($P<0.001$).

Significant interactions were found in both cohorts between patient sex and survival and between patient sex and age at diagnosis ($P<0.01$): females had better survival but tended to be older than males. A further significant negative interaction was found in the deceased patients' cohort between DHA quartile of residence and place of death ($P<0.01$). Quartile 1, comprising the districts with the lowest DCO registration ratios, had the smallest proportion of cases dying in the NHS and the largest proportion of cases dying at home, while the converse was true in quartile 4. The fit of both models improved when age at diagonsis was supplemented by the squared term (age at diagnosis squared),

**Table I** Proportion of registrations by 'death certificate only' (DCOs) at the Thames Cancer Registry. All cases, 1987–89, aged under 100 years at diagnosis. By age group at diagnosis and sex (odds ratios are relative to male cases aged under 40 years)

| | Male cases | | | Female cases | | |
|---|---|---|---|---|---|---|
| *Age group (years)* | *DCOs (%)* | n | *Odds ratios (95% CIs)* | *DCOs (%)* | n | *Odds ratios (95% CIs)* |
| Under 40 | 9.14 | 3566 | 1.00 | 7.00 | 4345 | 0.75 (0.64–0.88) |
| 40–64 | 17.59 | 20 554 | 2.12 (1.89–2.38) | 13.25 | 25 639 | 1.52 (1.35–1.71) |
| 65–74 | 22.63 | 25 603 | 2.91 (2.60–3.26) | 21.54 | 21 093 | 2.73 (2.43–3.06) |
| 75 and over | 32.42 | 29 765 | 4.77 (4.29–5.31) | 34.81 | 31 566 | 5.31 (4.78–5.81) |
| Total | 24.39 | 79 488 | 3.21 (2.88–3.58) | 23.27 | 82 643 | 3.01 (2.70–3.36) |
| *P* (trend) | | | <0.0001 | | | <0.0001 |

**Table II** Two year relative survival and percentage of DCO registrations for the 15 most common sites for cancer in males

| *ICD code (tumour site)* | *Survival* | *DCOs (%)* | n[a] |
|---|---|---|---|
| *Good survival* | | | |
| 186 (testis) | 93.10 | 2.36 | 1061 |
| 172 (skin) | 76.34 | 9.50 | 1053 |
| 161 (larynx) | 73.53 | 11.41 | 1209 |
| 188 (bladder) | 69.07 | 12.02 | 6282 |
| *Moderate survival* | | | |
| 185 (prostate) | 56.44 | 22.59 | 9971 |
| 202 (lymphoid and histiocytic tissue) | 48.66 | 22.94 | 2001 |
| 154 (rectum) | 48.30 | 16.62 | 3923 |
| 153 (colon) | 42.55 | 24.46 | 5703 |
| 189 (kidney and other unspecified urinary organs) | 42.39 | 21.50 | 1856 |
| 203 (multiple myeloma) | 33.10 | 32.52 | 1187 |
| 191 (brain) | 24.33 | 17.21 | 1482 |
| *Poor survival* | | | |
| 151 (stomach) | 13.95 | 30.19 | 5062 |
| 150 (oesophagus) | 11.70 | 24.82 | 2111 |
| 162 (lung) | 10.73 | 28.79 | 20 692 |
| 157 (pancreas) | 6.82 | 33.68 | 2586 |

[a]Survival is calculated for all cases except DCO cases.

**Table III** Two year relative survival and percentage of DCO registrations for the 15 most common sites for cancer in females

| *Tumour site* | *Survival* | *DCOs (%)* | n[a] |
|---|---|---|---|
| *Good survival* | | | |
| 172 (skin) | 84.07 | 7.77 | 1712 |
| 174 (breast) | 72.31 | 16.38 | 22 403 |
| 180 (cervix uteri) | 69.35 | 11.88 | 2686 |
| 182 (uterus) | 69.35 | 12.51 | 3117 |
| *Moderate survival* | | | |
| 188 (bladder) | 58.13 | 16.81 | 2498 |
| 202 (lymphoid and histiocytic tissue) | 47.41 | 21.34 | 1879 |
| 154 (rectum) | 47.17 | 19.18 | 3389 |
| 153 (colon) | 41.08 | 26.36 | 7239 |
| 183 (ovary and other uterine adnexa) | 35.57 | 21.45 | 4331 |
| 189 (kidney and other unspecified urinary organs) | 34.59 | 25.37 | 1088 |
| 191 (brain) | 26.79 | 22.83 | 1108 |
| *Poor survival* | | | |
| 151 (stomach) | 15.35 | 33.73 | 3217 |
| 150 (oesophagus) | 12.26 | 26.82 | 1633 |
| 162 (lung) | 9.89 | 30.22 | 9671 |
| 157 (pancreas) | 5.05 | 36.01 | 2777 |

[a]Survival is calculated for all cases except DCO cases.

**Figure 1** Standardised DCO registration ratios, Thames Cancer Registry 1987–89, by DHA of residence. All tumour sites combined with 99% confidence intervals.

**Table IV** Percentage distribution of all deaths and DCOs (in brackets) in each quartile by place of death. All percentages are based on quartile totals

| | Quartile | | | | |
|---|---|---|---|---|---|
| *Place of death* | *1* | *2* | *3* | *4* | *All* |
| Acute NHS | 44.43 | 46.72 | 49.08 | 52.73 | 48.32 |
| | (30.21) | (37.57) | (41.24) | (47.87) | (40.88) |
| Extra regional | 0.46 | 0.49 | 0.53 | 0.33 | 0.45 |
| | (0.73) | (0.73) | (0.95) | (0.43) | (0.67) |
| Home | 29.86 | 27.69 | 30.03 | 25.08 | 28.29 |
| | (44.93) | (39.19) | (38.65) | (31.09) | (37.14) |
| Hospice | 12.48 | 13.01 | 9.50 | 12.75 | 12.00 |
| | (10.18) | (10.03) | (8.55) | (11.52) | (10.35) |
| Independent | 2.95 | 3.45 | 1.11 | 1.72 | 2.32 |
| | (3.85) | (5.72) | (1.89) | (2.65) | (3.37) |
| Long-stay NHS | 2.44 | 1.86 | 1.53 | 2.64 | 2.13 |
| | (4.24) | (2.68) | (2.67) | (3.97) | (3.42) |
| Nursing home | 2.58 | 1.49 | 1.93 | 0.88 | 1.69 |
| | (4.04) | (2.05) | (2.79) | (1.10) | (2.24) |
| Oncology | 1.36 | 2.16 | 0.91 | 0.54 | 1.24 |
| | (0.71) | (1.07) | (0.36) | (0.33) | (0.57) |
| Other hospital | 0.52 | 0.53 | 2.87 | 0.48 | 1.05 |
| | (0.65) | (0.64) | (2.53) | (0.40) | (1.01) |
| Post-graduate | 0.17 | 0.24 | 0.51 | 1.03 | 0.56 |
| | (0.20) | (0.22) | (0.30) | (0.30) | (0.37) |
| Total deaths | 24 224 | 25 099 | 22 740 | 26 392 | 98 455 |
| % deaths | 100 | 100 | 100 | 100 | 100 |
| Total DCOs | 5373 | 6831 | 7301 | 11 052 | 30 557 |
| %DCOs | 100 | 100 | 100 | 100 | 100 |

signifying that the relationship between age at diagnosis and source of registration was non-linear.

Table VII shows that, within all quartiles, the odds of being registered as a DCO increase for patients dying in the private sector, hospices and at home. Odds also broadly increase by quartile (though odds are frequently higher in DHA quartile 3 than in DHA quartile 4). For patients dying in the private sector, the odds of being registered by DCO (relative to patients registered in DHA quartile 1, dying in the acute sector) were 2.74 in quartile 1, 3.24 in quartile 2, 4.03 in quartile 3 and 2.96 in quartile 4. For patients dying at home the corresponding figures were 2.87, 5.62, 9.27 and 5.97 respectively.

**Table V** A logistic regression model for registration by DCO. Top 15 cancers for men and women combined ($n$ = 134 927). All patients, alive and deceased

| *Variable* | *Odds ratio (95% CI)* | *Deviance difference* ($\chi^2$) | *d.f.* | P |
|---|---|---|---|---|
| DHA quartile of residence | | 3100.60 | 3 | <0.001 |
| Quartile | | | | |
| 1 | 1 | | | |
| 2 | 1.66 (1.27–2.17) | | | |
| 3 | 1.96 (1.51–2.56) | | | |
| 4 | 2.13 (1.63–2.79) | | | |
| Survival (%) | 0.96 (0.96–0.97) | 2031.84 | 1 | <0.001 |
| Age | | | | |
| At diagnosis (years) | 1.00 (1.00–1.01) | 6185.27 | 2 | <0.001 |
| At diagnosis squared | 1.00 (1.00–1.01) | | | <0.001 |
| Sex (females *vs* males) | 0.76 (0.63–0.92) | 96.01 | 1 | <0.001 |

**Table VI** A logistic regression model for registration by death certificate only (DCO). Top 15 cancers for men and women combined ($n$ = 98 455). Deceased patients only

| *Variable* | *Odds ratio (95% CIs)* | *Deviance difference* ($\chi^2$) | *d.f.* | P |
|---|---|---|---|---|
| DHA quartile of residence | | 3417.36 | 3 | <0.001 |
| Quartile | | | | |
| 1 | 1 | | | |
| 2 | 1.57 (1.47–1.68) | | | |
| 3 | 2.17 (2.03–2.32) | | | |
| 4 | 3.71 (3.48–3.95) | | | |
| Survival (%) | 0.99 (0.98–1.00) | 95.53 | 1 | <0.001 |
| Age | | | | |
| At diagnosis (years) | 1.04 (1.03–1.05) | 2607.69 | 2 | <0.001 |
| At diagnosis squared | 1.00 (1.00–1.01) | | | |
| Sex (females *vs* males) | 0.87 (0.82–0.92) | 152.76 | 2 | <0.001 |
| Place of death | | 3637.11 | 9 | <0.001 |
| Acute NHS hospital | 1 | | | |
| Extra regional | 3.34 (2.12–5.26) | | | |
| Home | 2.87 (2.65–3.10) | | | |
| Hospice | 1.35 (1.20–1.51) | | | |
| Independent hospital | 2.74 (2.26–3.32) | | | |
| Long-stay hospital | 2.89 (2.36–3.53) | | | |
| Nursing home | 2.39 (1.92–2.98) | | | |
| TCR area unidentified hospital | 2.73 (1.81–4.12) | | | |
| Oncology | 0.85 (0.57–1.26) | | | |
| Post-graduate hospital | 2.57 (1.25–5.30) | | | |

**Table VII** Multiple logistic regression model: deceased patients only. Interaction of place of death with DHA quartile of residence. Odds of being registered by DCO (relative to cases resident in DHA quartile 1 dying in the acute sector)

| | *Odds ratios* | | | |
|---|---|---|---|---|
| *Place of death* | *Quartile 1 (lowest DCO ratios)* | *Quartile 2 (second lowest DCO ratios)* | *Quartile 3 (second highest DCO ratios)* | *Quartile 4 (highest DCO ratios)* |
| Acute NHS hospital | 1.00 | 1.57 | 2.17 | 3.71 |
| Extra regional | 3.34 | 6.83 | 6.20 | 5.67 |
| Home | 2.87 | 5.62 | 9.27 | 5.97 |
| Hospice | 1.35 | 2.81 | 3.39 | 4.72 |
| Independent hospital | 2.74 | 3.24 | 4.03 | 2.96 |
| Long-stay hospital | 2.89 | 6.91 | 6.74 | 4.81 |
| Nursing home | 2.39 | 5.34 | 7.05 | 6.62 |
| TCR area, unidentified hospital | 2.73 | 5.84 | 10.67 | 11.68 |
| Oncology | 0.85 | 1.79 | 3.82 | 5.43 |
| Post-graduate hospital | 2.57 | 7.38 | 20.25 | 39.05 |

**Discussion**

DCOs accounted for a quarter of all registrations in the TCR between 1987 and 1989. This compares unfavourably with rates given by other registries in England and Wales, which ranged from 1.6% to 13.8%. The factors we found to be associated with DCO registration were: increasing age, decreasing survival, district of residence and place of death. Between 1987 and 1989, the over-65s accounted for 67.2% of all registered cases but 80.8% of DCO registrations. Age at diagnosis also interacts with survival time, so that the increase in DCO registrations with age is partly a function of shorter survival time. And survival time is, in turn, strongly correlated with tumour site.

The aim of this study was to ascertain factors associated with DCOs that might be amenable to registry intervention. We identified three factors which merit further investigation: tumour site, district of residence and place of death.

For all tumour sites, DCO rates are very high: for example, it is unlikely either that 16.6% of breast cancers or that 9.5% of skin cancers would be diagnosed only at death. Some tumour sites such as multiple myeloma appear to have disproportionately large numbers of DCO registrations, which suggests that some aspect of the treatment process or setting makes registration more difficult.

After adjusting for age and sex, there are significant variations in DCO ratios by district of residence. However, these do not take account of differences in tumour site and survival. Districts with high rates of poor-survival cancers might be expected to have higher DCO rates, as they would have less time to register cases in life. Indeed, the observed variations in district DCO ratios could be a function of inter-district differences in the burden of disease (Table IV). However, our multiple logistic regression models indicate that district differences in DCO rates persist, even after adjusting for differences in age, sex and survival time. Patients resident in the fourth quartile dying in the acute sector were 3.71 times more likely to be registered as DCO cases than their counterparts in the first quartile. In the cohort comprising deceased patients only, we were able to confirm that the strong association with DHA quartile of residence was independent of place of death. This is consistent with an observation reported in a previous study that the organisation of medical records departments appeared to affect the ability of clerks and researchers to retrieve notes (Vickers and Pollock, 1993).

However, nearly 41% of all DCO patients die in the acute NHS hospital sector, which suggests both a failure of ascertainment and a failure of DCO registration procedures in acute NHS hospital settings. More efficient registrations at NHS hospitals could dramatically reduce the proportion of DCOs in Thames.

Dying outside the NHS also increases the odds of being a DCO, so that in quartile 1 elevated odds ratios (relative to patients dying in the acute sector) were also found for patients dying in hospices (OR = 1.35), long-stay treatment centres (OR = 2.89), nursing homes (OR = 2.39) and private institutions (OR = 2.74). These results suggest that changing patterns of care will have serious implications for the ability of registries to ascertain cases both prospectively and retrospectively. Shorter lengths of stay and more day case and out-patient treatments may make it harder for registry clerks to retrieve notes and ascertain cases. It is to be hoped that the introduction of the minimum contract data set for cancer will counteract these developments (NHS ME Executive Letter, EL(92)95). Greater use of the private sector and hospices may also make registration more difficult and time-consuming for clerks attempting to retrieve retrospective information on cases ascertained by death certificate as neither the private sector nor hospices are bound by the minimum contract data set to provide information to the cancer registries.

*Conclusion*

OPCS and cancer registries should collect routine data on DCOs by site, district of residence and place of death. Cancer registration quality assurance programmes should use these measures to improve the efficiency of case ascertainment and registration programmes and to consider the implications of high rates of DCOs for cancer registration.

**Acknowledgements**
We wish to thank Ms Barbara Butland for statistical advice, the Thames Cancer Registry for providing data and SW Thames Regional Audit monies for funding the research.

**References**

BRESLOW NE AND DAY NE. (1987). *Statistical Methods in Cancer: The Design and Analysis of Cohort Studies*, 2 Vols. Lyon: IARC.

CHOW WH AND DEVESA SS. (1992). Death certificate reporting of colon and rectal cancers. *JAMA*, **267**, 3028.

GARDNER M AND ALTMAN D. (1989). *Statistics with Confidence: Confidence Intervals and Statistical Guidelines*. London: BMJ Publications.

HAKULINEN T, GIBBERD R, ABEYWICKRAMA K AND SÖDERMAN B. (1988). *A Computer Program Package For Cancer Survival Studies*, Helsinki: Cancer Society of Finland.

JENSEN OM, PARKIN DM, MACLENNAN R, MUIR CS AND SKEET RG. (1991). *Cancer Registration: Principles and Methods*, Lyon: IARC Scientific Publications.

OPCS. (1993). *Cancer Statistics: Registrations, 1987*. London: OPCS.

OPCS, CANCER RESEARCH CAMPAIGN. (1981). *Cancer Statistics: Incidence, Survival and Mortality in England and Wales*. London: OPCS, Cancer Research Campaign.

PERCY C, STANEK II E AND GLOECKLER L. (1981). Accuracy of cancer death certificates and its effects on cancer mortality statistics. *Am. J. Public Health*, **71**, 242–250.

POLLOCK AM AND VICKERS N. (1994*a*). The reliability of Thames Cancer Registry data on 673 colorectal cancer cases: the effect of the registration process. *Quality and Health Care*. (in press).

POLLOCK AM AND VICKERS N. (1994*b*). The impact on colorectal cancer survival of cases registered by death certificate only, implications for national survival rates. *Br. J. Cancer*, **70**, 1229–1231.

SAS INSTITUTE. (1990). *SAS/STAT Users' Guide, version 6*, 4th ed, Vol 1 & 2. Cary, NC: SAS Institute.

SILMAN AJ AND EVANS SJW. (1981). Regional differences in survival from cancer. *Commun. Med.*, **3**, 291–297.

SWERDLOW AJ. (1986). Cancer registration in England and Wales: some aspects relevant to interpretation of the data. *J. R. Stat. Soc.*, **A149** (2), 146–160.

THAMES CANCER REGISTRY. (1992). *Cancer in South East Thames 1987–1989*. Sutton, Surrey: Thames Cancer Registry.

VICKERS N AND POLLOCK A. (1993). Incompleteness and retrieval of case notes in a case note audit of colorectal cancer. *Quality Health Care*, **2**, 170–174.

WILSON S, PRIOR P AND WOODMAN CB. (1992). Use of cancer surveillance data for comparative analyses. *J. Public Health Med.*, **14**, 151–156.


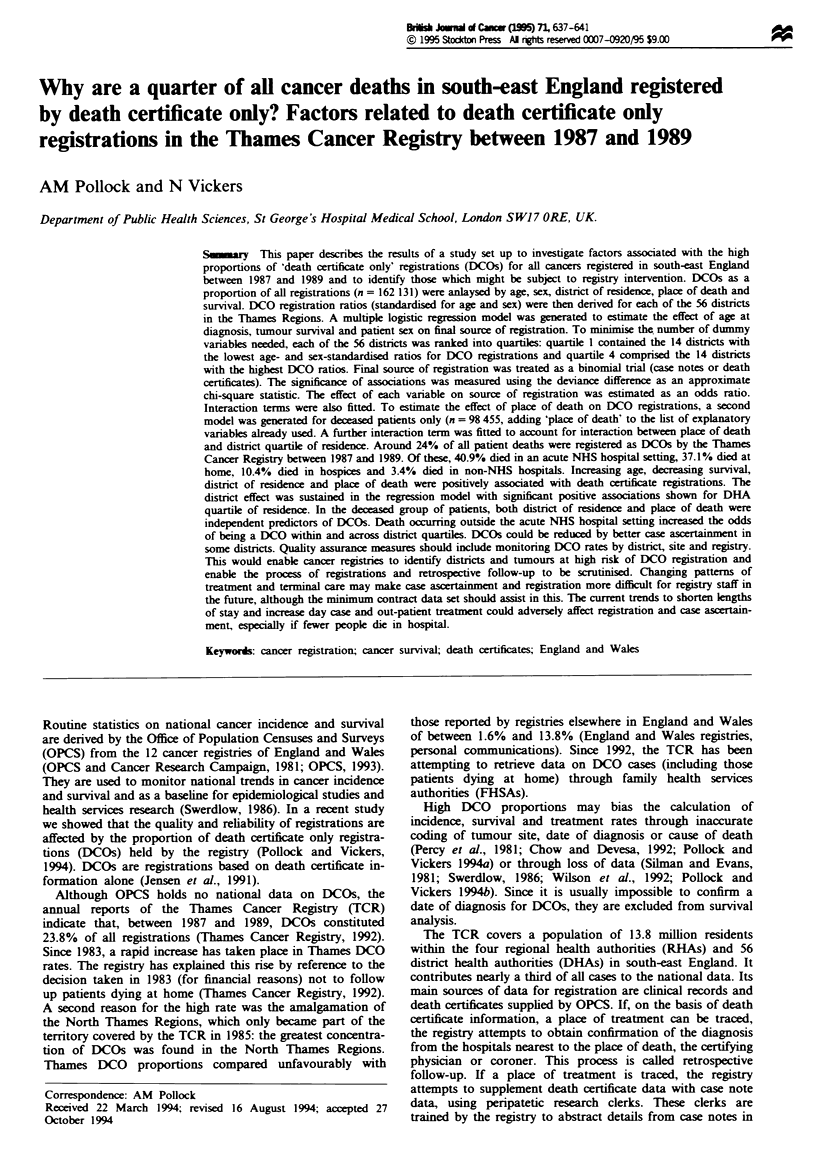

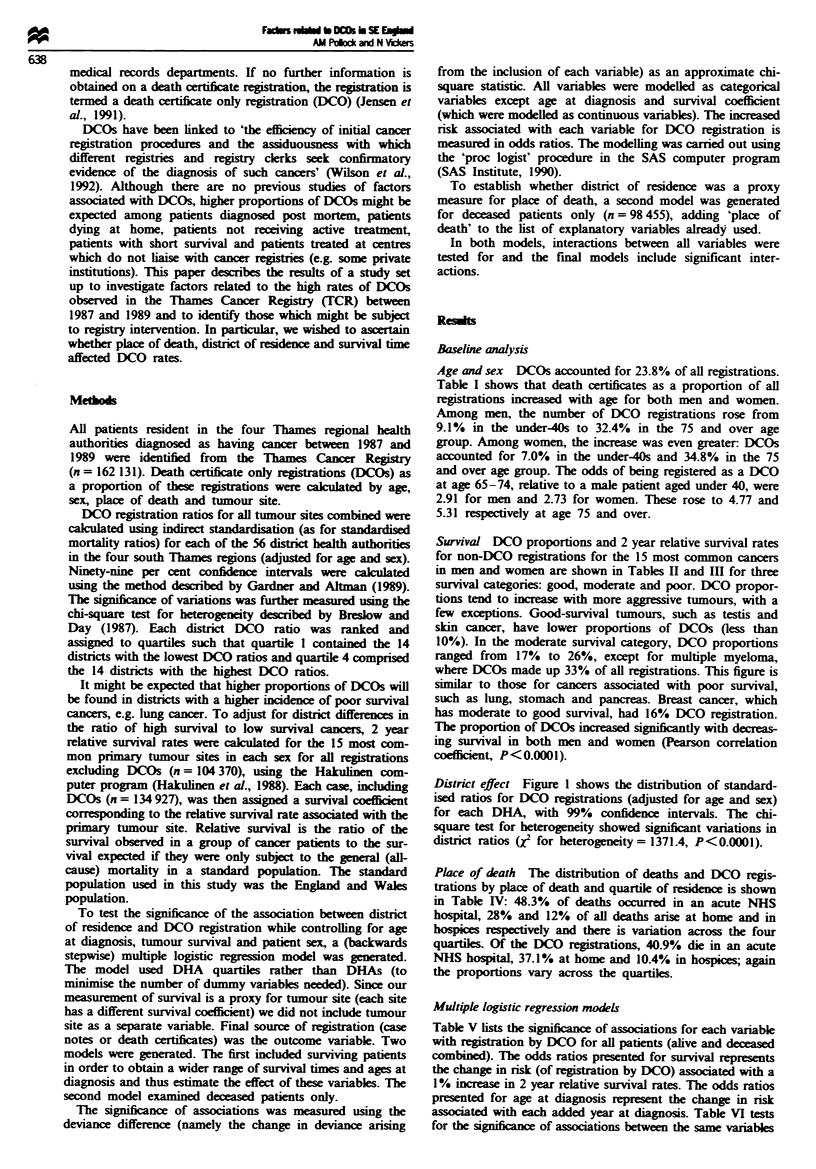

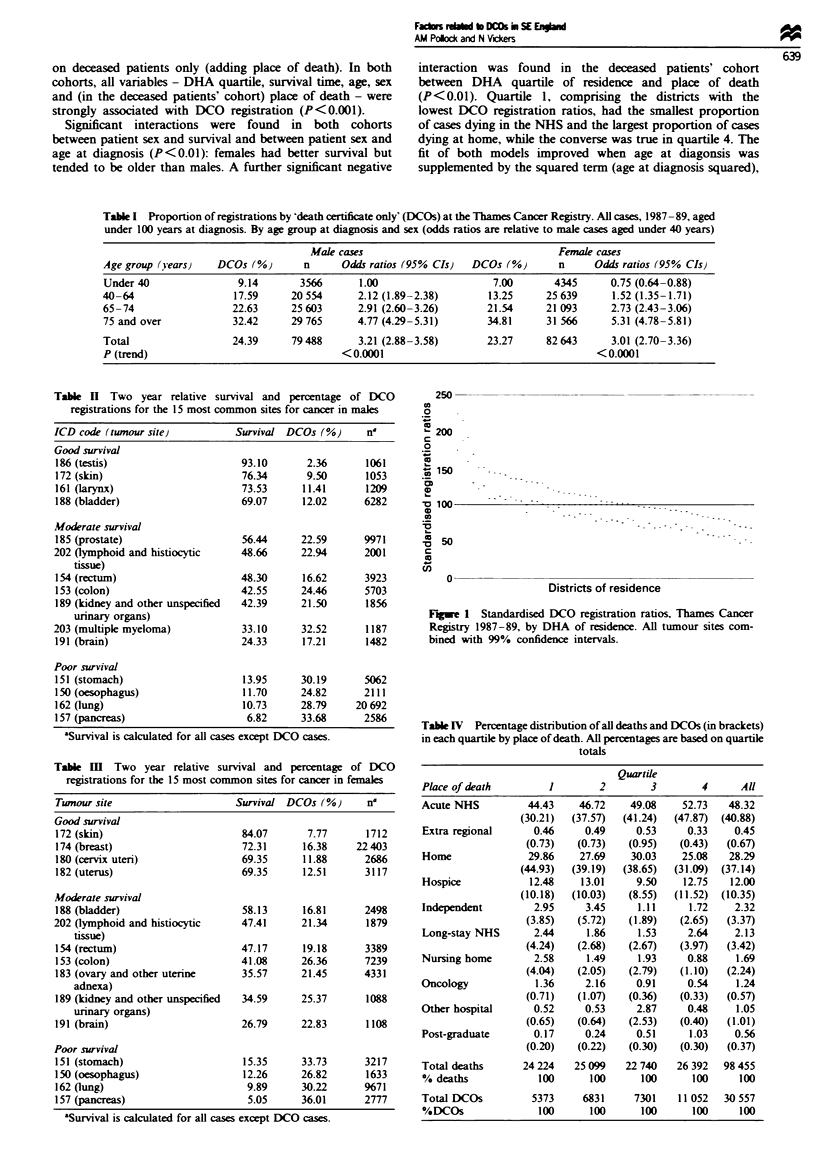

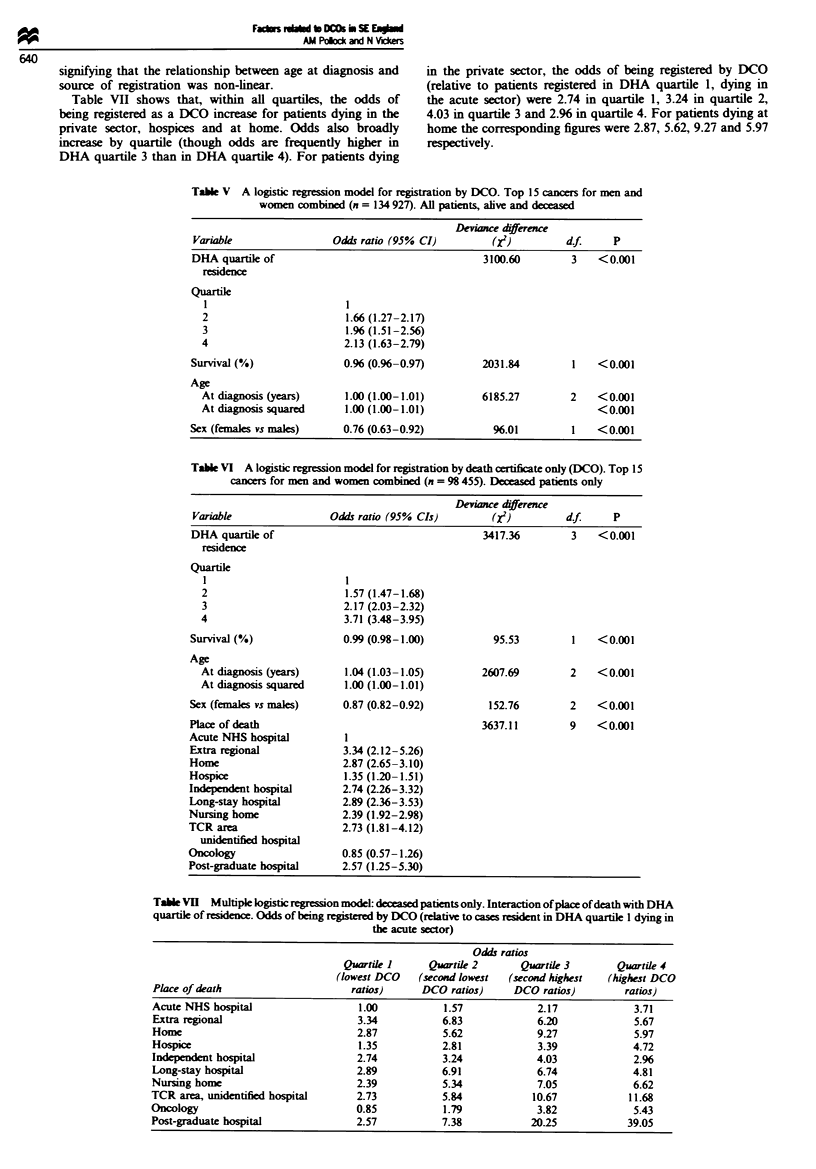

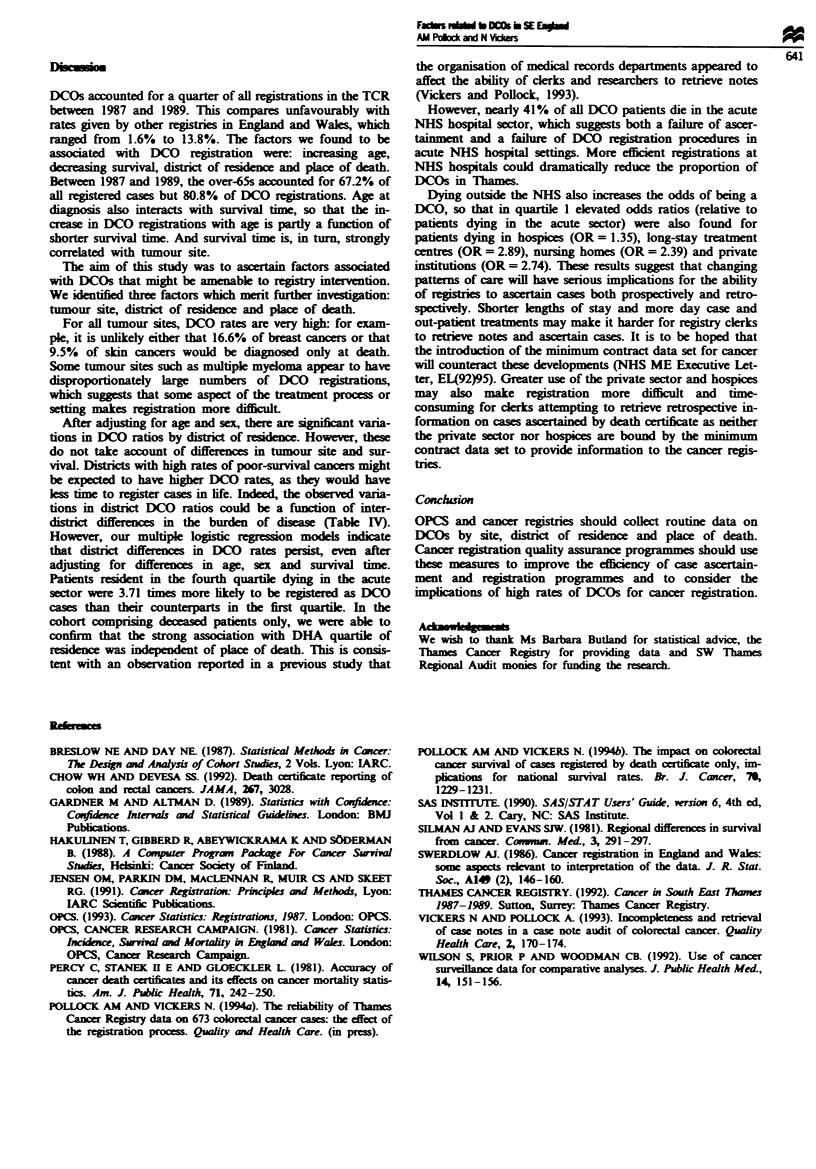


## References

[OCR_00813] Chow W. H., Devesa S. S. (1992). Death certificate reporting of colon and rectal cancers.. JAMA.

[OCR_00838] Percy C., Stanek E., Gloeckler L. (1981). Accuracy of cancer death certificates and its effect on cancer mortality statistics.. Am J Public Health.

[OCR_00860] Silman A. J., Evans S. J. (1981). Regional differences in survival from cancer.. Community Med.

[OCR_00871] Vickers N., Pollock A. (1993). Incompleteness and retrieval of case notes in a case note audit of colorectal cancer.. Qual Health Care.

[OCR_00876] Wilson S., Prior P., Woodman C. B. (1992). Use of cancer surveillance data for comparative analyses.. J Public Health Med.

